# Hospital and Institutional News

**Published:** 1919-11-22

**Authors:** 


### HOSPITAL AND INSTITUTIONAL NEWS.

### QUEEN CHARLOTTE'S HOSPITAL.

If we may judge from the statements made to us of the procedure being followed recently in this hospital the pupil nurses who had the courage to attach their names to a letter confirming the state of affairs as they then existed, and others, have been reduced to a condition approaching terror by the methods pursued towards individuals. We are informed that an investigation by one of the medical staff was ordered by the committee. At the hour named the pupil nurses were assembled in the corridor and there remained for some considerable time, but nothing happened, and ultimately they were dispersed to duty. Queen Charlotte's Hospital can never have the administrative efficiency, or continuously give the best of treatment and accommodation to those who there seek instruction, until a change of atmosphere takes place, and all the devices of a seminary are rendered impossible of adoption within its walls. The secret of its failure seems to be an establishment which has got out of hand and out of gear. The incidents which have happened, and the line adopted by some of those attached to the institution, demonstrate, that it would probably be to the advantage of the public and the group of nurses affected, if the institution were closed for, say, six months, and the organisation completely overhauled. Meanwhile young women are clamouring in great numbers for instruction in midwifery, and we suppose it is possible that they will continue to clamour, whatever the conditions of service, on the ground that they must get a certificate, and that the time of training is short.

### COUNTY HOSPITAL'S SENIOR SURGEON HONOURED: FORTY-TWO YEARS ON THE STAFF.

Monday afternoon will be memorable in the history of the General Hospital, Northampton, which was founded in 1743 and mainly rebuilt in 1904. The great interest taken in the proceedings on this memorable day was evidenced by the fact that no fewer than 160 of the subscribers, out of a total of 260, were present in person. Dr. G. H. Percival, the senior surgeon, one of the most highly respected members of the medical profession in Northampton, on his resignation of office after forty-two years' devoted service, was presented with a cheque for £400 by Sir Charles Knightley, Bart., on behalf of the governors, in the presence of a notable gathering of influential people representing all classes, including the patients, who are keen supporters of the county hospital. Mr. R. V. Loder, the chairman, was emphatic that they all owed Dr. Percival an enormous debt for the way he had stuck to the hospital during the war. His intention had been to resign earlier, but when war broke out he decided to keep on the arduous work which he carried out so willingly and extremely well. Sir Charles Knightley, in presenting the gift on behalf of the subscribers, said: "Dr. Percival first became associated with the hospital in 1864 when he entered it as a pupil, and three years later was appointed assistant house surgeon. Dr. Percival next went to Guy's Hospital, and, after practising in Derbyshire for a time, in 1878 he applied for the post of surgeon to the Northampton County Hospital, and very fortunately for the hospital he succeeded in being appointed. He had held the position ever since, a period of nearly forty-two years. Only one member of the medical staff in 176 years had held office for a longer period than Dr. Percival. This was Dr. Kerr, physician to the hospital from 1763 to 1824." We should like to have the names and particulars of the members of other hospital staffs, if any there be, who have remained on the staff of their hospital for a longer period than sixty-one years. Truly, Dr. Kerr's was a remarkable achievement.

### EVER FAITHFUL AND TRUE.

The developments in treatment and the advancement made in medicine, surgery, orthopædics, and many branches of the healing art have been momentous indeed, and Dr. Percival has the honour of having been a most devoted friend of the county hospital, 'who, by his energy and enthusiasm, carried the governors and county with him, and secured momentous developments, including the practical rebuilding of the hospital in 1904. Subsequently an up-to-date and thoroughly efficient pathological laboratory was added, both steps of great importance, which tended to increase the efficiency of the treatment, to extend the area of supporters, and materially aid the curative results which the staff were able to secure for the patients. An old supporter and friend of the County Hospital, who was confined to the house by sudden illness, telegraphed: "Am greatly disappointed not to be present. Have known Dr. Percival for a lifetime. No surgeon could render more whole-hearted service than his to the County Hospital for decades of years; ever faithful and true to his hospital and professional responsibilities has Dr. Percival been in all things. May God's blessing sustain him in his retirement." The reports of the proceedings which we have so far received are incomplete, but we hope to return to this interesting ceremony and to give some of Dr. Percival's reminiscences in an early number. It is interesting to add that Dr. Percival announced that he was going to spend the gift he had received that day in the purchase of a motor-car, that he might have the pleasure of moving about and seeing the world at his will and pleasure.

### PHYSICAL EDUCATION.

The recent correspondence upon this subject in the *Times* should serve to draw attention to a very urgent need in this country. At present we have no standard British system of physical education, and, excellent though the various systems are—originated mainly in other countries and adopted by us at various times and under various circumstances—yet it is obvious that, to achieve any real efficiency in this work, one single determining principle must guide our efforts. A Department of Physical Education is suggested, but, whatever the precise organisation, the need is for direction and leadership, and it is time that the Ministry of Health or the Ministry of Education, or both, took the matter in hand. It is not the training of the individual or the few which must be perfected, but physical education in its broadest and most comprehensive aims. "If the nation faces the task of physical education fairly it will realise that, while the physical condition of the present generation is an urgent and ever-present problem, the physical condition of the next is in our hands, and we should take every means possible to conserve the nation's boyhood, realising that physical vitality during youth is always a great reservoir for middle-age efficiency."

### A CENSUS OF CRIPPLES.

The Presidential address delivered at the annual meeting of the British Orthopædic Association brought forcibly before us the fact that this country will for many years be faced with the remote results of wounds and disease incurred in the war zones. Among others, there will be the survivors of some 50,000 wearers of artificial limbs, who will call for no little care, and, in view of future practice, the ultimate results of these cases should receive the closest attention. Also we have to learn the true application to civil work of the war-time surgery of fractures, and to watch and elaborate the wonderful beginnings which were made in operative treatment of disabilities resulting from spastic paralyses. In Germany, before the war, a census of cripples was undertaken and reported upon by Biesalski, and proved to be of the greatest value in concerted surgical development. At the moment we have in this country a relatively incomplete knowledge of the cases among us, and we heartily agree with the President, Mr. E. Muirhead Little, in his suggestion "that a census providing full details" should be made with as little delay as possible.

### THE PATHOLOGIST'S STATUS.

The circumstance that, at our greater hospitals, the pathologist has as a rule no official place on the medical committee has roused Professor Browning, on leaving the Middlesex Hospital for the Chair of Bacteriology in Glasgow University, to address a request to the Governors of the hospital that a change might be inaugurated with the appointment of his successor in office. This exclusion, where it exists, is obviously a custom dating from the time when true scientific medicine was in its very early infancy, and the changed modern circumstances must be obvious to all when it is realised that some of the most famous leaders of medical research are to be found at the head of pathological departments. The greatest advances have almost invariably benefited, if not resulted, from the closest co-operation between the physician at the bedside and the physician in the laboratory, and if anything tending to hinder such united work still exists it is the first duty of those in authority to remove it.

### THE STUDENTS' RAG.

The medical students of St. Bartholomew's and of University College Hospital in their friendly rivalry to capture and recapture the Bart's gun have, like the students of King's College, London, in their baptism of Mr. Pussyfoot, the abolitionist, with a bottle of Bass, reminded London that it is a University city. While we must be careful to defend the right of free speech, especially on the part of those whose opinions one may hold to be detestable (for their opinions are the only ones which it is a virtue to tolerate), the affair, but for a regrettable accident duly apologised for by the King's College students, seems to have been conducted with good humour. It is therefore absurd to regard it seriously. It needs neither formal attack nor defence, but is a youthful escapade which the victim himself will probably be good humoured enough to smile at. Since writing the above, however, we regret to learn that Mr. Johnson's eyesight is in danger from a missile thrown at him. That missile was not part of the programme, but the programme gave an opportunity to the wretch who threw it, and for that reason those who organise these rags have a heavy responsibility.

### NEW ANTHRAX CASES.

We read that inquiry is being made into the cause of a number of cases of anthrax supposedly associated with the use of certain shaving-brushes. As the result of the investigation by the City Bacteriologist, a consignment of brushes from Japan is reported as being infected with the organism. Specimen samples from that consignment had been provisionally reported upon as showing six further cases out of nine brushes examined. The Ministry of Health, in conjunction with local authorities concerned, are dealing energetically with this matter, a procedure more particularly necessary in that the consignment of brushes mentioned cannot possibly account for some of the human cases which have developed, and, in our own experience, with some of them there is no apparent connection with shaving-brushes at all, but with the usual process of unloading hides. It would appear we are again faced with a small outbreak, due probably to the same deficient measures at the ports of origin to which so much attention was quite recently drawn.

### INTERNATIONAL PUBLIC HEALTH.

The Office International d'Hygiène Publique, an official body which was constituted in 1907 by a large number of the Governments of the world to investigate international questions relating to public health, has recently concluded one of its sittings in Paris. One of the most urgent questions of the kind with which the Office International d'Hygiène Publique has to concern itself, is raised by the present epidemic of typhus in Poland. The League of Red Cross Societies recently dispatched a medical Commission to Poland, consisting of Colonel H. S. Cumming, of the United States Federal Public Health Service; Dr. G. S. Buchanan, of the British Ministry of Health; Dr. A. Castellani, (Italy); and

Medécin-principal Visbecq, of the French Army Service de Santé. The Commission investigated the measures of precaution and treatment which are being taken on the Eastern Fronts in the occupation of the Polish Armies and in the interior, by the new Polish Ministry of Health. Both the military and the civil organisations were found to be enlightened and efficient, but hampered in their work by a shortage of food and hospital and household material. The situation, aggravated as it is by the constant movement along the Eastern Frontier of Poland of refugees and prisoners, is deemed by the Commission to present serious risk of the spread this winter of the epidemic towards the Western parts of Europe. The League of Red Cross Societies while expressing itself willing to aid the situation by all means in its power feels that the need is greater than it can hope to supply, and it has accordingly brought the report of the medical commission to the notice of different Governments which are in a position to help by the supply of material and personnel, and has urged those Governments to render all the assistance in their power. The Office International d'Hygiène Publique at its recent session associated itself fully with these representations in the belief that measures against typhus in Poland are of the greatest importance to safeguard other countries against serious epidemics of this most formidable disease. At the same session the Office International d'Hygiène Publique had under consideration its own future relationship to the League of Nations (which is required by the terms of its written constitution to interest itself in international questions of public health), and decide to place itself unreservedly at the disposal of the Governments which form the League for discussion and consideration of the way in which its work may best be combined and extended under the direction of the League.

### A THREATENED FIASCO AT CARDIFF.

The allegations lately made against the dangerous condition of the toilers at King Edward VII. Hospital, Cardiff, have been referred to a small subcommittee who will shortly report. But we refer to them meantime because they gave an opportunity to Mr. Leonard Rea, the general superintendent, to explain the position to which he is reduced by overwork, insufficient devolution of duties, and, we believe, unsatisfactory accommodation. The matter in principle is simple enough. King Edward VII. Hospital is but half way through a Herculean task. It has built and extended. It has paid a good deal for bricks and mortar. It has become, at least potentially, one of the great hospitals of the day. But the work is incomplete. The income is too small, the expenses of maintenance are very heavy; the waiting-list is still over 1,000. The last statement is a scandal to Wales. The result is that the general superintendent must be set free from routine work in order to develop a policy equal to the situation. He is doubly in need of his freedom since the death of Colonel Bruce-Vaughan, who was the statesman in charge of the hospital's policy. Death has robbed the hospital of its old leader. Much of his work has devolved on Mr. Rea. If he is not allowed freedom to take the task in hand, the ambitions to which the committee is pledged in honour and by past performance will be imperilled, for a half-finished task is the worst of fiascos. More than this, to shackle Mr. Rea with routine at this critical moment when he needs all his faculties for the higher branches of his work, is to risk the loss of his health also. Those on the spot fail to realise the seriousness of the situation, both on its public and personal side. We do not envy them their responsibility in this matter.

### CHANGE OF OCCUPATION.

Amongst the appointments required and advertised for in a daily paper is one of £1,000 a year, sought by a doctor who is "fed up with a lot of night work." The advertiser is in very large general practice and is at present working eighteen hours a day; his age is twenty-eight, and he has to his credit four years' active service on all fronts. He took three first prizes in his course at a London Hospital, and now desires the annual sum named with prospects "in a job where his scientific knowledge will be an asset." We suppose it is as difficult for the general practitioner returning from the war to settle down into the daily routine as it is for the layman, and equally difficult for each to avoid using slang in serious speech.

### HOSPITALS AND COMPENSATION CASES.

Voluntary hospitals stand up to be shot at in respect of compensation cases. It is in the interests of the patient, the employer, the insurance company, and the defending party, in street and other accident cases, that the voluntary hospital carries out its work—it lessens the damage and makes a valuable saving, as a rule, to the efficiency of the individual. In a letter to *The Evening Standard*, Dr. Savage brings out these points, following on his public protest against the guinea fee. The case in which the question arose cost the Metropolitan Hospital approximately £50, and this institution, £30,000 in debt, does not stand to benefit to the extent of one penny as a result of the litigation ensuing. Yet its medical officer has to leave his very exacting duties and spend a considerable part of the day in a Court of Law, receiving for his personal service the sum of one guinea. Dr. Savage states that an offer was made to furnish all the medical information the Court could need without fee or payment, provided that the solicitor engaged would also work gratuitously. The justice of such a bargain would not, of course, stand scrutiny for a moment, but the spirited protest in this case may very likely lead to reconsideration of a system which bears heavily upon the medical profession.

### AFFRAY WITH A CHINAMAN.

The presence of Asiatics at the Poplar and Stepney Sick Asylum has more than once led to complications which have caused Dr. Spurrell, the medical superintendent, to advocate a separate institution for these patients. An alarming incident lately again occurred when a Chinaman, unable to speak or understand English and whose mentality therefore was difficult to diagnose, suddenly became maniacal, and made a desperate assault upon his own throat. He was prevented, said Dr. Spurrell, by the pluck of Nurse Hopkins, who tackled him alone in spite of his powerful build, and held him, knife in hand, till Nurse May Horan and Nurse Agnes Lawler came to her aid. Their readiness and devotion, said Dr. Spurrell, averted a disaster, and a letter of congratulation and thanks has been voted to the three.

### THE PLIGHT OF THE NEURASTHENIC PATIENT.

The lack of hospitals for ex-Service men continues, and is felt perhaps most severely by neurasthenic cases. To remedy the local deficiency the West Riding War Pension Committee is pressing the Ministry of Pensions, which is taking Oulton Hall, near Leeds, for an unspecified purpose, to use it for neurasthenic patients. The present hospital in Stainbuck Lane, Chapel Allerton, Leeds, is much too small for the whole of Yorkshire; and there is, despite the present hospital, a waiting-list of one hundred such patients from the West Riding alone. Nor does the treatment provided at Harrogate meet the need, because the patients have to live in lodgings, a doubtful plan which is also so expensive that the cost of a new hospital would almost be provided by the sum spent upon their lodgings. What is wanted, it may be surmised, is a central institution at Harrogate, where the treatment itself is good, and some subsidiary hospitals, like Oulton Hall, to meet the local needs of what is, after all, our largest county.

### A STATE VIEW OF OUR PUBLIC HEALTH SERVICES.

The multiplication of officials gives sometimes, because of the societies wherein they group themselves, an opportunity for the rest of us to learn how bureaucracy, in the technical sense, appears to its members. What do they think of the State machine? It is well to know, and we regret for this reason that no full report is to hand of an address lately given by Dr. F. H. Scroggie, assistant county medical officer of health to the Lanarkshire branch of the National Association of Local Government Offices. Dr. Scroggie was advocating the unification of local health services, and the reduction of overlapping. He sought to cover the whole ground, and to reveal by way of criticism of existing organisation what the service ought to be over which the Ministry of Health should preside. We think that men at the periphery are likely often to take a more statesmanlike view than doctrinaires at Whitehall, and advise our readers to keep an alert eye on similar local addresses.

### IS THERE HOPE OF BETTER GAS?

The hospital housekeeper or matron in London, and probably elsewhere, has been in despair because of the wretched quality of gas for cooking purposes, and now that the cold weather is coming on, unless there is an improvement, sitting-rooms and dressing-rooms in many institutions will be dangerously cold, especially to patients who are undressed for examination or treatment. While the war was on it was understood that some of the most valuable constituents of gas had to be diverted for the production of ammunition, and now, twelve months after the armistice, things are as bad as ever. It is whispered that the threatened coal strike has delayed a decent supply, but now, with things brightening in the industrial world, there should be no longer an excuse. At last a little hope is given to the long-suffering public through news of a system of supply which, we understand, the gas companies are agreeable to adopt. It is a system under which quality is paid for rather than quantity, and the unit will be one of heat rather than capacity. The calorific value of the gas supplied is to be continuously measured and shown on a recording calorimeter, and all that will be necessary for the calculation of the amount due from the consumer will be to multiply the number of cubic feet registered on the gas meter by the declared gross calorific value of the gas per cubic foot.

### THE ADMISSION OF PAYING PATIENTS.

The West of England Eye Infirmary at Exeter has had long to wait for the surrender of its buildings by the military, but part of them are being set in order at once. Notwithstanding difficulties, however, the infirmary has done some good and even progressive work, for last year it arranged to receive patients who were able to pay something, but were alike ineligible for free treatment and unable to afford the doubtful luxury of a nursing home. The plan proved advantageous to the hospital. This new departure is the last to take place during the term of Mr. A. C. Roper, who has resigned the post of senior surgeon after thirty-seven years, and has been, unlike some medical officers, a pillar also of the committee. During the war, Mr. Roper carried on his work without help, and his retirement is a serious loss to the institution.

### THIS WEEK'S DRUG MARKET.

The upward tendency of prices has become more pronounced, and in some departments business is active. Considerable interest continues to centre in menthol, Japanese peppermint oil, and camphor, the two former having again advanced in price substantially. Menthol is in fact now about six times the figure that used to be regarded as normal. The price of potassium bromide is firmly maintained; the small quantities that have been imported from Germany have barely affected the market. Several of the synthetic drugs have moved upwards in value, these being antifebrin, hexamine, and phenazone; it is not improbable that phenacetin will follow suit. A small supply of ergot of rye is now available, and is selling at extreme prices. The good demand for eucalyptus oil continues, and the value has an upward tendency. The value of ipecacuanha is firmly maintained. The upward movement in the price of lemon oil has ceased for the moment; orange oil is dearer. Business has been done in senega root at higher prices.

